# Glial Cells in the Fish Retinal Nerve Fiber Layer Form Tight Junctions, Separating and Surrounding Axons

**DOI:** 10.3389/fnmol.2018.00367

**Published:** 2018-10-10

**Authors:** Lidia Garcia-Pradas, Corinna Gleiser, Andrea Wizenmann, Hartwig Wolburg, Andreas F. Mack

**Affiliations:** ^1^Institut für klinische Anatomie und Zellanalytik, Universität Tübingen, Tübingen, Germany; ^2^Institut für Pathologie und Neuropathologie, Universität Tübingen, Tübingen, Germany

**Keywords:** claudin, teleost, myelin, oligodendrocyte, Müller cell, regeneration, *Astatotilapia burtoni*

## Abstract

In the retina of teleost fish, cell addition continues throughout life involving proliferation and axonal growth. To study how this is achieved in a fully functioning retina, we investigated the nerve fiber layer (NFL) of the cichlid fish *Astatotilapia burtoni* for components that might regulate the extracellular environment. We hypothesized that growing axons are surrounded by different cell structures than signal conducting axons. Using immunohistochemistry and freeze fracture electron microscopy we found that the endfeet of Müller cells (MCs) expressed aquaporin-4 but not in high densities as in mammals. The presence of this water channel indicates the involvement of MCs in water homeostasis. Remarkably, we discovered conspicuous tight junctions in the retinal NFL. These tight junctions formed branching strands between myelin-like wrappings of ganglion cell axons that differed morphologically from any known myelin, and also an elaborate meshwork on large membrane faces between axons. We speculated that these tight junctions have additional functions than solely facilitating nerve conductance. Immunostainings against the adaptor protein ZO-1 labeled the NFL as did antibodies against the mammalian claudin-1, 3, and 19. Performing PCR analysis, we showed expression of claudin-1, 3, 5a, 5b, 9, 11, and 19 in the fish retina, claudins that typically occur at brain barriers or myelin. We could show by immunostains for doublecortin, a marker for differentiating neurons, that new axons are not surrounded by the myelin-like wrappings but only by the endfeet of MCs. We hypothesize that the tight junctions in the NFL of fish might contribute to the separation of an extracellular space around axons facilitating conductance, from a growth-promoting environment. For a functional test we applied Evans Blue dye to eye cup preparations which showed a retention of the dye in the NFL. This indicates that these remarkable tight junctions can indeed act as a diffusion barrier.

## Introduction

In the vertebrate nervous system, neurons are usually separated from circulating cells and compounds by structures such as the blood-brain barrier and blood-cerebrospinal fluid barrier. In the case of the eye these are termed the blood-ocular barrier (Hosoya and Tomi, 2005) which consists of the outer barrier located at the tight junctions of the pigment epithelial cells (Rizzolo et al., 2011) and the inner barrier at the endothelial cells if blood vessels are present. We were interested in the interface between the retina and the vitreous humor in a system where function and retinal growth have to be accomplished at the same time (see below). The inner limiting membrane is the border of the inner retina to the vitreous and consists of a basal lamina paved with the endfeet of Müller cells (MCs) but does not contain a junctional barrier. The blood-aqueous humor barrier is located mainly at the ciliary body.

In the innermost cell layer of the vertebrate retina, the axons of ganglion cells (GCs) give rise to the optic nerve projecting to the brain. These axons are surrounded by myelin formed by oligodendrocytes. In mammals, myelination begins mostly at the lamina cribrosa, and GC axons running in the nerve fiber layer (NFL) within the retina are accompanied only by the processes of MCs or astrocytes.

The retina of anamniotes, especially of teleost fish, has some characteristics that are distinctly different from the mammalian retina. These include a highly regular mosaic of photoreceptors and other retinal elements, retino-motor movement as a mechanism of light adaptation, and continuous and mostly life-long growth of the retina (Engström, 1963; Maier and Wolburg, 1979; Burnside and Dearry, 1986; Yazulla and Studholme, 2001; Mack, 2007; Bejarano-Escobar et al., 2014). The continuous growth implies cell addition from specialized growth zones or progenitor cells (Müller, 1952; Fernald, 1989; Mack and Fernald, 1993; Lenkowski and Raymond, 2014). Among the added cells are retinal GCs which send their axons to the brain, and therefore new growth cones have to navigate through the existing NFL.

The NFL however, is also penetrated by the processes and endfeet of Müller (glial) cells playing an essential role for the physiology of the retina: Müller cell endfeet are particularly important for ionic homeostasis in the retina since they express high levels of potassium and water channels, providing the basis for a mechanism referred to as K^+^ siphoning. This is essential for proper neuronal signaling, yet a growth-promoting environment for axon elongation has to be maintained within the functioning retina.

In this study, we investigated the fiber layer of the retina in the cichlid fish *Astatotilapia burtoni* to elucidate structural components of the interface between the neural retina including the NFL and vitreous. We hypothesized that cellular structures directly surrounding growing axons differ from the surroundings of existing axons. The retina of *A. burtoni* has been extensively studied previously, in addition to behavioral, social, and genetic aspects of this species (Mack and Fernald, 1995; Renn et al., 2008; Fernald, 2012; Mack and Tiedemann, 2013). This species has the advantage of rapid and extensive growth over several years. We first used several histological approaches including ultrathin section and freeze-fracture electron microscopy, and subsequently molecular methods to reveal unusual tight junctions in the NFL.

## Materials and Methods

### Animals

For this study, we used eyes of the cichlid fish *A. burtoni* bred in our own colony. All procedures were performed according to governmental guidelines and were approved by local authorities (Regierungspräsidium Tübingen). We used animals of either sex between 3 months and 1 year of age with a standard length of 3–5 cm. For the removal of the eyes, animals were anesthetized with MS222 and killed by cervical section.

### Immunohistology

For immunohistological stains, excised eyes were fixed in 4% paraformaldehyde after removal and processed as previously described (Mack et al., 2004). Briefly, eyes freed of the lens and sclera were rinsed and cryoprotected in 30% sucrose before frozen and sectioned on a cryostat at 18 μm. Primary antibodies were applied on sections overnight at 4°C after preincubation in normal goat serum. See **Table 1** for the detailed information on antibodies used. After three washes in PBS, secondary antibodies were applied for 1.5 h, rinsed again and coverslipped in Mowiol. In some stains we applied the nuclear stains Sytox Green (excited at 488 nm) or DRAQ5 (excited at 633 nm; both from Thermo Fisher Scientific). Sections were examined on a LSM510 confocal microscope (Zeiss, Oberkochen, Jena, Germany), using laser excitations at 488, 543, and 633 nm in sequential scans with appropriate filter sets. The images were captured with ZEN 2009 software (Zeiss), linear contrast and brightness adjustments and assembling of image plates were carried out with Adobe Photoshop CS4.

**Table 1:** List of antibodies used.

Primary Antibody	Specificity/Immunogen	Source (catalog number)	Host	Dilution
Anti-claudin-1	C-terminal of human/mouse claudin-1	Thermo Fisher Scientific (71-7800)	Rabbit	1:100
Anti-claudin-3	C-terminal of mouse claudin-3	Thermo Fisher Scientific (34-1700)	Rabbit	1:100
Anti-claudin-19	C-terminal of mouse claudin-19	A gift from Dr. M. Furuse	Rabbit	1:100
3A10 anti-neurofilament-associated antigen	Neurofilament associated protein from chick spinal cord	Iowa Hybridoma Bank	Mouse	1:100
Anti-ZO-1	Aa 334-634 of human ZO-1	Thermo Fisher Scientific (33-9100)	Mouse	1:100
Anti-myelin basic protein	Aa 82-87	Abcam ab7349	Rat	1:125
Anti-Doublecortin	C-terminal of human doublecortin	Santa Cruz Biotechnology sc-8066	Goat	1:100
Anti-proliferating cell nuclear antigen (PCNA)	Rat PCNA fusion protein	DAKO M0879	Mouse	1:300
Anti-aquaporin-4	Aa 244-323 at C-termial of human AQP4	Santa Cruz Biotechnology sc-20812 (H80)	Rabbit	1:100
Anti GFAP	Porcine GFAP	Santa Cruz Biotechnology sc-58766	Mouse	1:100
Anti-proteolipid protein (PLP-1)	N-terminal of PLP1	Thermo Fisher Scientific (PA5-40788)	Rabbit	1:100
**Secondary Antibody**		**Source**	**Host**	**Dilution**
Anti-rabbit Alexa 488		Thermo Fisher Scientific	Goat	1:400
Anti-mouse Alexa 546		Thermo Fisher Scientific	Goat	1:400
Anti-rabbit Alexa 546		Thermo Fisher Scientific	Goat	1:400
Anti-goat Alexa 488		Thermo Fisher Scientific	Donkey	1:400
Anti-mouse Alexa 546		Thermo Fisher Scientific	Donkey	1:400
Anti-rat Alexa 546		Thermo Fisher Scientific	Goat	1:400

### Electron Microscopy

For electron microscopy, retinal and brain tissues were dissected and fixed in 2.5% glutaraldehyde (EM grade) for 2 h at room temperature. For freeze-fracturing, specimens were cryoprotected in 30% glycerol and snap-frozen in nitrogen slush (-210°C). Subsequently, they were fractured in a Balzer’s freeze-fracture device (BAF400D; Balzers, Liechtenstein) at 5 × 10^-6^ mbar and -150°C. The fracture faces were shadowed with platinum/carbon (3 nm, 45°) for contrast and carbon (30 nm, 90°) for stabilization of the replica. After removal of the cell material in 12% sodium hypochlorite, the replicas were rinsed in double-distilled water several times and mounted on Pioloform-coated copper grids.

For ultrathin sections specimens were postfixed in 1% OsO_4_ in PBS, and dehydrated in an ethanol series (50, 70, 96, and 100%). Dehydration was completed by acetone, followed by propylene oxide. The 70% alcohol was saturated with uranyl acetate for contrast enhancement. After infiltration, specimens were embedded in Epoxy medium (Sigma Aldrich, Darmstadt, Germany). Ultrathin sections (60 nm) were prepared on a Leica Ultramicrotome (Leica, Bensheim, Germany) and mounted on pioloform-coated copper grids.

Freeze-fracture replicas and ultrathin sections were examined on a LEO 912AB transmission electron microscope (Carl Zeiss, Oberkochen, Germany).

### RT-PCR

For the molecular biology experiments, the retinas were prepared under RNase-free conditions. The surrounding pigmented epithelium, where the presence of TJs is well documented, was carefully removed to avoid contamination with non-NFL tight junctions. Brains were separately collected to be used as reference sample in the PCR analysis. The tissue was collected in Precellys^®^ Ceramic Beads Kit 1.4/2.8 mm tubes (VWR Life Science Competence Center, Erlangen, Germany) containing QIAzol Lysis Reagent (Qiagen, Hilden, Germany) and immediately stored at -80°C. After thawing on ice, the tissue was disrupted in a Minilys Personal Homogenizer at 500 rpm for 10 s (Bertin Instruments, Montigny-le-Bretonneux, France). After incubating the homogenate for 5 min at room temperature, 100 μl of gDNA Eliminator Solution (Qiagen) was added. The homogenate was transferred to a MaXtract High Density Tube (Qiagen), supplemented with chloroform and centrifuged at 12.000 ×*g* for 30 s. The upper, nucleic acid-containing, aqueous phase was pipetted in a fresh tube and total RNA isolation was automated in the QIAcube (Qiagen) using the RNeasy Plus Universal Mini Kit (Qiagen) following manufacturer’s protocols. The RNA concentration of all samples was measured with the Qubit 2.0 Fluorometer (Thermo Fisher Scientific, Darmstadt, Germany). RNA was converted into cDNA using the QuantiTect Reverse Transcription Kit (Qiagen) following manufacturer’s description. PCR was performed with 2 μl samples of the RT-reaction, 0.2 μM of the forward and reverse primers, 0.2 mM dNTP, 2 mM MgCl_2_ and 1.25 U Taq polymerase (VWR Life Science Competence Center). All primers (see **Table 2**) were specifically designed for *A. burtoni* predicted mRNA-sequences, which were retrieved from National Center for Biotechnology Information (NCBI), using DNASTAR Software. PCR was performed for 35 cycles: 30 s denaturation at 95°C, followed by 40 s annealing at 62°C, and 1 min elongation at 72°C. The PCR products were analyzed with standard electrophoresis on 1.5% agarose gels at 100 V, stained with peqGREEN (VWR Life Science Competence Center) and photographed under UV illumination with a gel documentation system (E-Box VX2, Vilber Lourmat, Eberhardzell, Germany). The size of each PCR product was estimated by using a peqGold 100 bp DNA Ladder (VWR Life Science Competence Center).

**Table 2:** Primers used for RT-PCR analysis.

Accession No.	Sequence	Primer sequence	Primer (bp)	Amplicon
XM_005939627	Claudin-1-like	for 5′ GCGCGGAGAGGCCAACATTT 3′	20	459 bp
		rev 5′ CCCGCCAGGTAGCAGCACACT 3′	21	
XM_005932576	Claudin-3-like	for 5′ ACCGGCGTGGCTCTTGGACT 3′	20	432 bp
		rev 5′ TTTAAACTCGGGCGGGAACTCG 3′	22	
XM_005924382	Claudin-5a-like	for 5′ GTTGCGTGCGGGTTACCTATGTGG 3′	24	421 bp
		rev 5′ CAGCGCCGATCTCCCTCTTCTTG 3′	23	
XM_005928973	Claudin-5b-like	for 5′ GAGCGCCTTCATCGATTCCAACA 3′	23	414 bp
		rev 5′ GCCGCCCAGCCCACATAGATT 3′	21	
XM_005940343	Claudin-9-like	for 5′ CCCGCATTGCTCGCATTTCTGG 3′	22	277 bp
		rev 5′ GGTGCCCTGCGTGGACTGCTCTC 3′	23	
XM_005926028	Claudin-11-like	for 5′ TGGGCGCATTGTGTCATCTCTA 3′	22	451 bp
		rev 5′ GGTTGCTGCCTCCCTGTTTG 3′	20	
XM_005934508 XM_005934509 XM_005934510	Claudin-19-like	for 5′ TCTGGCGTTGGGTGGCTGGATT 3′ rev 5′ CGGGACTTGGTGGCTGGGTTGT 3′	22 22	315 bp

### Evans Blue Experiments

To test for a possible barrier function of the TJs located in the NFL, experiments with Evans Blue dye were carried out. Evans Blue has previously been used for permeability experiments (Saria and Lundberg, 1983) including the retina (Nicchia et al., 2016). Eyes were removed from fish (ca 3.5 cm SL), the cornea opened and the lens removed. Approximately 10 μl of a 2% Evans Blue dye in PBS solution were pipetted into the empty space left by the lens. Eyes were incubated in PBS at room temperature for either 5 or 20 min, and washed thoroughly afterward. Then, the retina was isolated by removing the sclera and quickly sliced with a tissue chopper (Sorvall, Newton, CT, United States). Slices were transferred to glass-bottom Petri dishes (FD 35–100, WPI) and imaged immediately on an inverted stand confocal laser scanning microscope (LSM 5 Exciter, Zeiss, Oberkochen, Germany) using a 10× objective and an He/Ne laser with 546 nm excitation and a longpass filter at 560 nm. As controls for the diffusion of the dye through retinal tissue, we incised the eyecup preparation from two sides of the ciliary periphery to the center leaving the optic nerve head intact. Application of Evans Blue and tissue processing was then performed as described above. For quantification, we recorded the fluorescence intensity profile along a transect line through optical sections of retinal slices from the inner limiting membrane to the outer nuclear layer, or in the case of the control preparations from the cut edge of the retina horizontally in the inner nuclear layer. Along this line, we measured the fluorescence intensity within an area of 1 μm^2^ at two locations 30 μm apart corresponding to the NFL and the inner plexiform layer in the experimental slices, using the Zeiss ZEN software. The ratio of these values from the same slice indicate the rate of diffusion of the dye.

For the experiments with Evans Blue we used a total of seven fish. From each animal one eye was prepared for the short incubation time (5 min) and the other eye for the long incubation period (20 min). For quantification, at least three measurements from each slice, and from at least three slices from different eyes and treatments were preformed.

## Results

**FIGURE 1:**
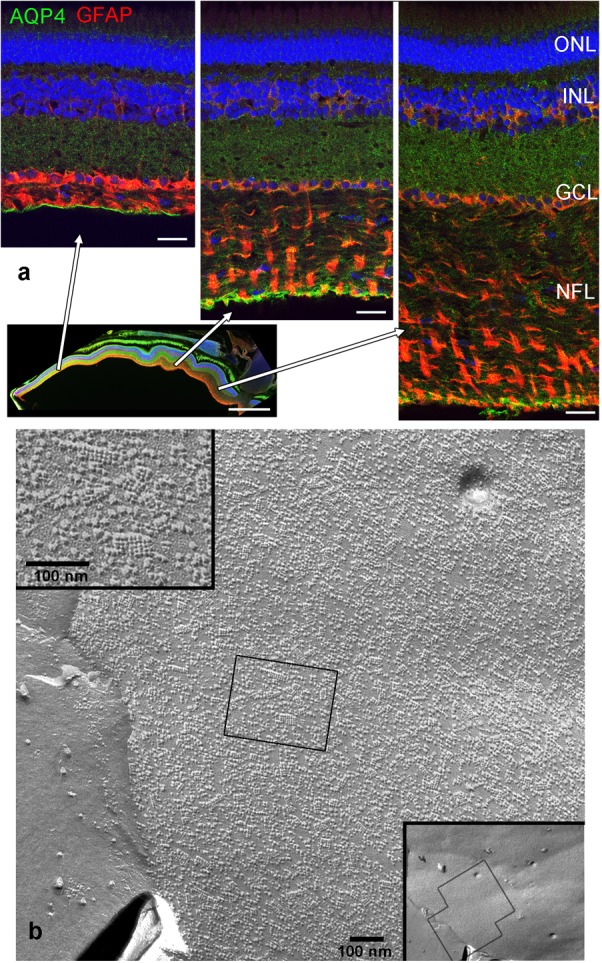
Nerve fiber layer (NFL) in the retina of an adult cichlid fish (SL 6 cm). **(a)** Sections from different retinal regions as indicated by the arrows starting at the overview image demonstrate the increase in thickness of the NFL from the peripheral region (left) to the region close to optic nerve head (right). Müller cell processes labeled with GFAP (red) pass through the NFL and form endfeet at the interface to the vitreous. Müller cells express aquaporin-4 (AQP4, green) in higher concentrations at these endfeet. Nuclei are labeled with Draq5. **(b)** Freeze fracture replica of Müller cell endfeet showing some orthogonal arrays of particles known to consist of AQP4, left inset. The right inset shows an overview, the black boxes indicate locations of higher power views. ONL, outer nuclear layer; INL, inner nuclear layer; GCL, ganglion cell layer; scale bars in **(a)** are 20 μm, in the overview image 500 μm, in **(b)** 100 nm.

In this study, we focused on the glial elements in the NFL in the cichlid fish retina known for its life-long growth. First, to investigate the glial structures in the NFL, we stained the retina for GFAP and the water channel aquaporin-4 (AQP4). Mammalian MCs express AQP4 in particularly high densities in the endfeet. As expected, we found AQP4 staining along Müller cell fibers, with slighly higher intensity at the endfeet but with less pronounced polarity compared to mammals (**Figure 1a**). The growth of the fish retina is reflected by the thickening of the NFL: In larger fish, the NFL close to the optic nerve head is thicker than the rest of the retinal layers together due to the addition of new GCs in the retinal periphery, but the NFL is relatively thinner in smaller conspecifics. In either case it is radially penetrated by the processes of MCs which form endfeet facing the vitreous and covering completely the basal lamina known as the inner limiting membrane (**Figure 1**). Since AQP4 is known to form orthogonal arrays of particles (OAPs) we performed freeze-fracture electron microscopy. Few OAPs were indeed found in freeze fracture replicas (**Figure 1b**), however in much lower densities than they occur in the mammalian retina, consistent with AQP4 immunostains. Other regions did not show any OAPs, neither in the retina nor in the brain.

**FIGURE 2:**
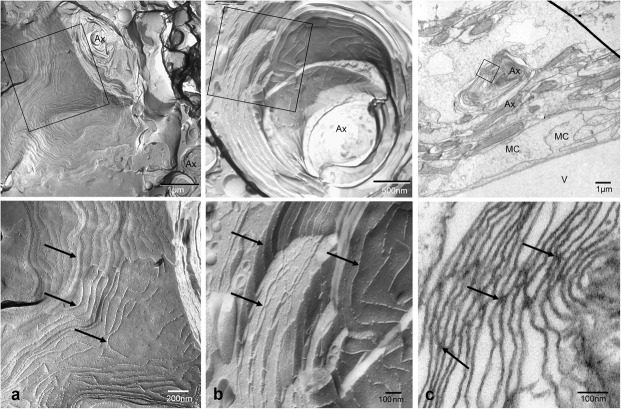
Tight junctions in loose wrap myelin in freeze fracture and ultrathin section electron micrographs of the retinal NFL. Tight junction strands (arrows) do not run parallel to the axon in varying distances and patterns, and between wrapping layers **(a,b)**. Tight junctional contacts between lamellae could be verified in sections **(c)**. The top panel shows overviews, the square frames indicate areas shown at higher magnifications in the bottom panel. V, vitreous humor; MC, Müller cell endfeet; Ax, Axon. Scale bars as indicated.

**FIGURE 3:**
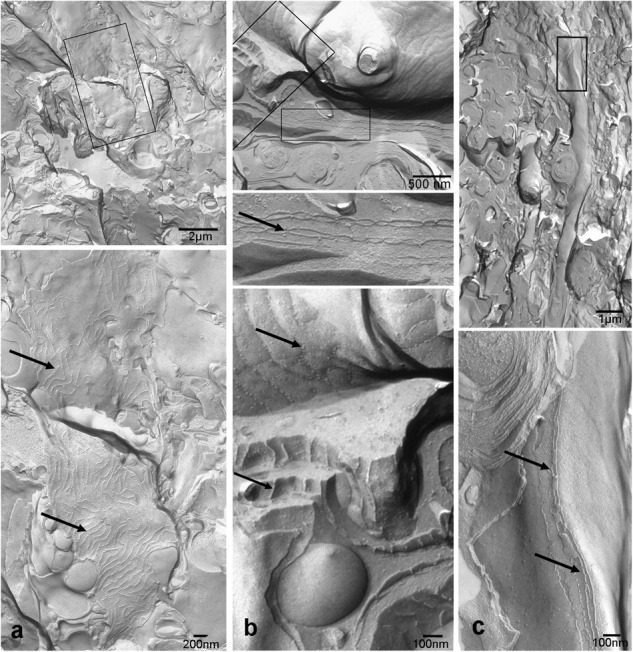
Tight junctions are not restricted to direct axonal wrappings but occur on membranes of larger processes **(a)**, or surrounding several axons **(b)**. In contrast, myelin in optic tectum depicted in **(c)** shows thin lamellae and few tight junction strands (arrows) oriented in axonal direction. Top panels represent overviews, higher magnifications areas marked by black frames are shown below. Scale bars as indicated.

Both histological approaches, however revealed two striking features that were further analyzed: (i) nuclear counterstains revealed many cells in the NFL (**Figure 1**) that did not stain for GFAP which makes it unlikely that they belong to astrocytes; and (ii) extensive membrane areas with numerous tight junction (TJ) strands were found in freeze fracture EM (**Figure 2**). Expanding this analysis we found that many of the elaborate TJs belonged to membranes surrounding axons in the NFL (**Figure 2a**). This myelin-like ensheathment of axons showed less densely packed lamellae with relatively thick cytoplasmic regions compared to textbook myelin as found in the optic tectum of the fish brain (**Figure 2b**, compare **Figure 3c**). Myelin in the fish NFL has previously been reported (Wolburg, 1980) and has been described as ‘loose wraps’ (Münzel et al., 2012). The TJ contacts between myelin lamellae could also be identified in ultrathin sections (**Figure 2c**) which also confirmed the irregular form of myelin-like ensheathment. In addition, cell membranes in between axonal fibers revealed also complex and sometimes discontinuous tight junction strands (**Figures 3a,b**). These did not belong to immediate axonal wrappings but to sheet-like processes in between groups of axon bundles. Most strikingly, the tight junction pattern surrounding axons in freeze-fracture preparations differed substantially from thin-layered, ‘regular’ myelin (**Figure 3c**).

**FIGURE 4:**
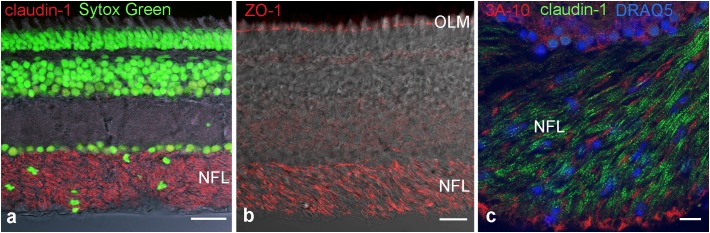
Immunohistochemical localization of tight junction proteins in cryosections of the retina. Anti-claudin immunoreactivity is clearly present in the NFL **(a,c)**, ZO-1 is present also at the outer limiting membrane. In **(c)** axons are labeled for the neurofilament- associated protein 3A-10. Nuclear stains clearly show the presence of cell nuclei in NFL. OLM, outer limiting membrane. Scale bars are 20 μm in **(a,b)**, 10 μm in **(c)**.

To identify molecular components of these TJ we used immunocytochemistry and PCR. First we used antibodies against the most common tight junction markers, namely the adaptor protein ZO-1 and the most ubiquitous member of the claudin family, claudin-1 to confirm the presence of TJ in the NFL (**Figures 4a,b**). Staining for claudin-1, and the neurofilament-associated protein 3A-10 revealed the localization of junctions in between nerve fibers (**Figure 4c**). These stains in combination with nuclear stains showed that axons are free of claudin-1 and the abundance of nuclei in the NFL in-between axons. These cell bodies were never positive for GFAP (cf. **Figure 1**) and decreased in density with the thinning NFL toward the retinal periphery. They most likely belong to cells forming TJs in the myelin-like wraps, Antibody binding for the adaptor protein ZO-1 was not only detected in the NFL, but also in the OLM (**Figure 4c**). This structure at the interface between Müller cell microvilli and the subretinal space was never positive for any of the claudins tested, indicating that ZO-1 is associated with a different junctional complex at this location.

Since more than 50 claudin genes have been identified in teleost fish (Loh et al., 2004; Baltzegar et al., 2013), we probed for selected potential candidates that may occur in the retinal NFL. The reason for selecting these claudins were, besides the ubiquitous claudin-1, that claudins-3 and -5 occur on blood-brain barrier forming endothelial cells, claudin-9 is related to the claudin k reported to be expressed in the zebrafish optic nerve. Claudin-11 in CNS, and claudin-19 in the PNS have been shown to occur in myelin. We therefore designed primers for these predicted *A. burtoni* claudin gene sequences with likely expression, specifically for claudin-1, claudin-3, claudin-5a, claudin-5b, claudin-9, claudin-11, and claudin-19 and performed RT-PCR experiments (for primers see **Table 2**).

**FIGURE 5:**
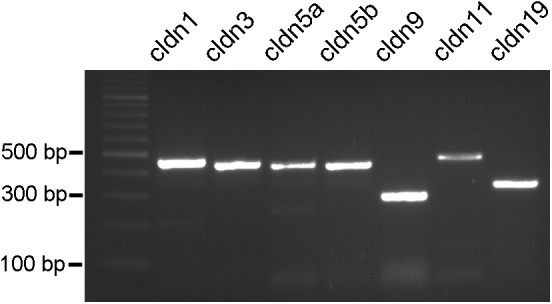
PCR products from cichlid fish retinal tissue. Clear single bands can be seen for claudin-1, claudin-3, claudin-5a, claudin-5b, claudin-9, claudin-11, and claudin-19 at the expected bp locations.

PCR conditions were optimized for all primer pairs using a temperature gradient and different MgCl_2_ concentrations. Furthermore, brain tissue was used as a reference tissue (data not shown). The PCR products resulted in single bands in agarose gels with the expected sizes for all of the claudins tested (**Figure 5**). These results were reproduced several times with at least five different samples.

**FIGURE 6:**
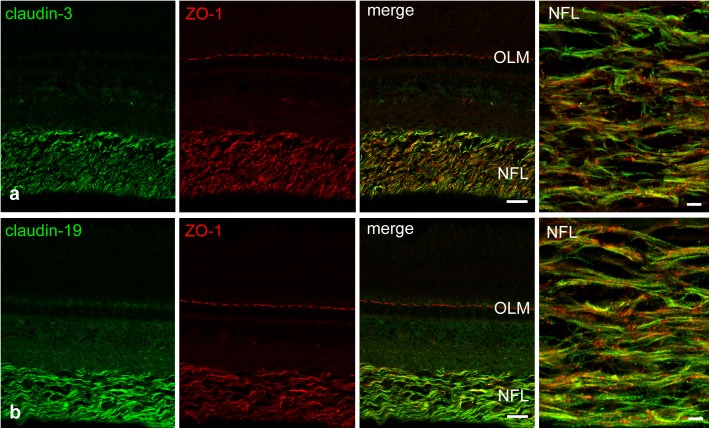
Double-immunostains for the tight junction proteins (green) claudin-3 **(a)** and claudin-19 **(b)** and for the adaptor protein ZO-1 (red, **a,b**). The nerve fiber layer (NFL) shown at high magnification in the right images, is strongly positive for both claudin antibodies. Immunoreactivity for ZO-1 was also detected in the NFL, as well as in the outer limiting membrane (OLM). In the NFL, claudin and ZO-1 stains largely overlap with the claudins showing more continuous label strands often but not always running parallel to nerve fibers. Scale bar 20 μm for low power, 5 μm for high power images.

We attempted to stain and localize further members of the claudin family by immunocytochemistry. Antibody reactivity against claudin-3 (**Figure 6a**), and claudin-19 (**Figure 6b**) was strongly present in the retina and largely restricted to the NFL. Punctate staining and short continuous strands could be observed running often but not always in the direction of axons. Immunostainings using antibodies against claudin-4, claudin-5, claudin-8, claudin-10, and claudin-16 were also carried out. However, none of these antibodies generated against mammalian claudins showed any tight-junction specific immunoreactivity in cichlid fish retinal sections (data not shown).

**FIGURE 7:**
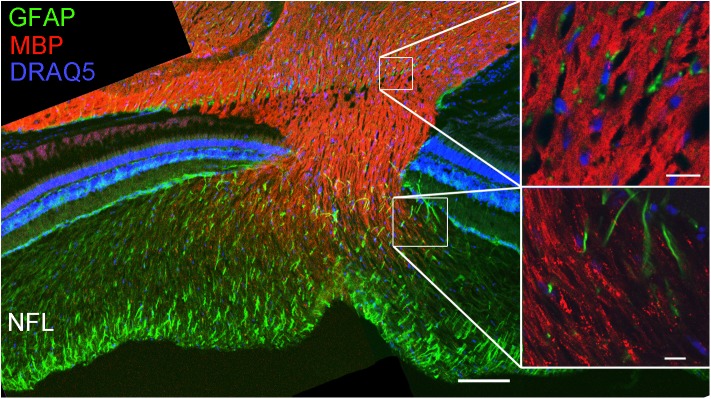
Loose wraps in the NFL lack myelin basic protein. Staining retinal sections through the optic nerve head demonstrates that the optic nerve is positive for myelin basic protein (MBP) but this reactivity decreases in the optic nerve head and is absent in the NFL. The high magnification images on the right show that the MBP immunoreactivity is homogeneous in the optic nerve (top) but discontinuous and punctate in the transition zone to the NFL. Scale bar 100 μm for the overview image, and 10 μm for the high power images.

For further characterization of NFL axon wrappings, we used an antibody for myelin basic protein (MBP). The staining showed that MBP is present in the optic nerve starting in the optic nerve head but no immunoreactivity was detected in the NFL (**Figure 7**), demonstrating a clear difference between loose wraps and regular myelin. We also probed for the proteolipid protein 1 (PLP-1), a typical component of mammalian myelin, and found that cells and nerve fiber wrappings in the NFL and in optic nerve head were negative for PLP-1, although immunoreactivity was present on Müller cell endfeet (data not shown).

**FIGURE 8:**
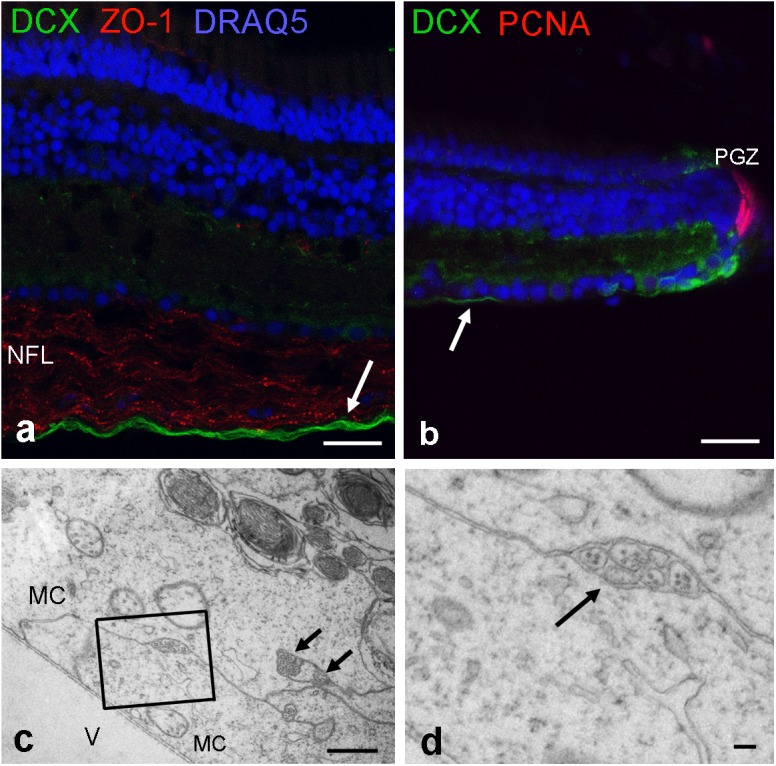
Newly formed axons are located on the innermost NFL. **(a)** Labeled by antibodies for doublecortin (DCX; green), new axons are found close to the inner limiting membrane. **(b)** These DCX axons originate from maturing GCs next to peripheral growth zone (PGZ) where proliferating cells are labeled by antibodies for proliferating cell nuclear antigen (PCNA). Tight junctions in the NFL are labeled by ZO-1. **(c,d)** Electron micrographs reveal that unmyelinated nerve fibers are separated from the vitreous (V) and surrounded by Müller cell endfeet (MC). New axons are indicated by arrows in **(a–c)**. Scale bars 20 μm in **(a,b)**, 1 μm in **(c)**, and 200 nm in **(d)**.

Next, we used an antibody for doublecortin (DCX), a marker for differentiating neurons to label new axons. Indeed cell bodies next to the peripheral growth zone were positive for DCX, and so were their axons. A double-label of DCX and ZO-1 revealed that newly generated axons run in the innermost region of the NFL, an area devoid of ZO-1 stain (**Figures 8a,b**). Thus, axons of newly generated GCs are not ensheathed by myelin-like wrappings but surrounded by Müller cell endfeet. This could be verified in ultrathin sections (**Figures 8c,d**).

**FIGURE 9:**
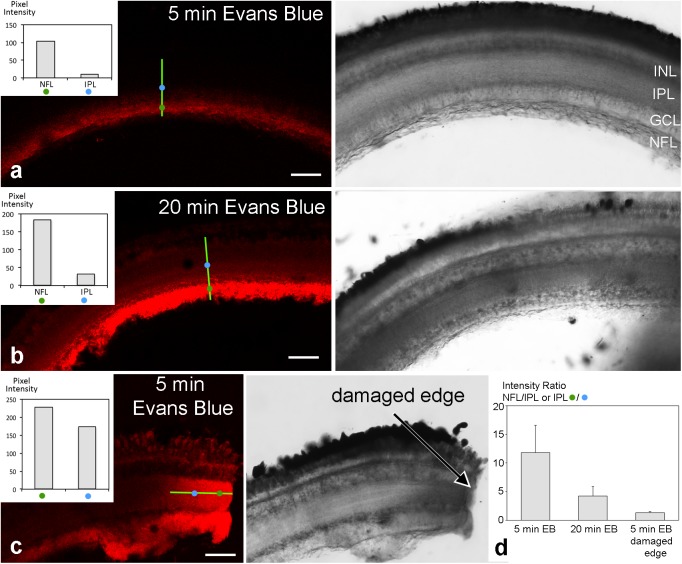
Unfixed retinal slices after eyecup treatment with Evans Blue. Confocal images are shown on the left, transmitted light images of the corresponding areas on the right. Fluorescence intensity was measured at two locations of 1 μm^2^ indicated by the blue and green circles 30 μm apart on a transect line (bright green) and are shown in the insets. After 5 min incubation time with Evans Blue dye, fluorescence is largely limited to the NFL **(a)**. After 20 min incubation time, some dye has diffused to the inner layers of the retina **(b)**. However, a sharp drop in fluorescence intensity remains from the NFL to inner and outer retinal layers. In contrast, in control preparations where eyecups had been incised prior to dye application, the dye diffused more readily from the cut end (arrow) into all retinal layers **(c)**. In **(d)**, the intensity ratios of individual measurements from locations as indicated in **(a–c)** are compared. A ratio of 1 would mean the same intensity at both measurement locations and unobstructed spread of the dye. The values >>1 for the experimental slices are consistent with the hypothesis that the NFL forms a diffusion barrier from the vitreous to the inner retinal layers. Error bars are SD (*n* = 13 for experimental and *n* = 9 for control sections). INL inner nuclear layer; IPL, inner plexiform layer; GCL, ganglion cell layer; NFL, nerve fiber layer. Scale bars 50 μm.

To test for a possible barrier function of the tight junctional structures in the retinal NFL, experiments were performed on excised fish eyes: after removing the lens, Evans Blue dye was pipetted into the empty eye cup. This preparation was incubated with the dye for different time periods (5–20 min), afterward slices were quickly prepared and imaged immediately. On the retinal slices of the cichlid fish eye which was incubated for 5 min with Evans Blue dye, immunofluorescence signal was largely restricted to the NFL revealing a sharp drop of fluorescence intensity (**Figure 9a**). Slices incubated for 20 min with Evans Blue dye showed an uneven gradient diffusion of the dye to inner layers of the retina but even after this longer incubation time, a sharp intensity drop could be seen at the ganglion cell layer (**Figure 9b**). In control experiments, Evans Blue was allowed to diffuse into the retina by an incision into the eye. At the cut end of such a slice, a linear diffusion gradient was found (**Figure 9c**). This resulted in a fluorescence intensity ratio of about 1.3 after 5 min Evans Blue incubation for the indicated locations 30 μm apart from each other. This ratio was much higher (11.8) in experimental slices with the same incubation period for locations in the NFL and inner nuclear layer (30 μm distance; **Figure 9d**). This indicates that the NFL can act as a diffusion barrier between the vitreous and the inner retinal layers.

## Discussion

Investigating the retinal NFL for components of the interface between the retina and the vitreous humor we discovered the exceptional structure and composition of TJ between the membranes of glial cells in the fish NFL. These TJ were found between membrane layers surrounding axons reminiscent of myelin, and also on sheet-like membrane faces in an interrupted meshwork pattern. In the mammalian retina, myelination of ganglion cell axonal fibers begins generally at the papilla of the optic nerve with some exceptions with few myelinated fibers like in rabbits (Reichenbach et al., 1988). Axons in the NFL are surrounded by processes of MCs, or astrocytes which occur only where blood vessels are present (Schnitzer, 1985). In the fish retina, however, retinal ganglion cell axons are wrapped by cellular sheaths (Wolburg, 1980; Easter et al., 1984; Münzel et al., 2012) termed ‘loose wraps’, different from typical CNS myelin.

We confirmed this difference by the comparison with myelin in the optic tectum where TJs run parallel to the nerve fiber. We demonstrated the expression of distinct claudin proteins by immunocytochemistry and PCR analysis, more specifically we found immunoreactivity for claudin-1, -3, and -19 in the NFL, and gene expression of claudin-1, 3, -5a, -5b, -9, -11, and -19. With the exception of pigmented epithelial cells which were carefully removed, no other cell type is known to possess TJ in the completely avascular cichlid fish retina. The immunostains for ZO-1 support the finding of TJs in the retinal NFL. Although ZO-1 is not an intrinsic component of TJs, it contains a PDZ domain that binds actin filaments at the COOH-terminal region, functioning as cross-linkers between TJ strands and actin filaments (Itoh et al., 1999). ZO-1 in the retina is also localized at the outer limiting membrane, consistent with previous observations, which localized this adaptor protein in the adherens junctions between MCs and the outer segments of photoreceptors (Campbell et al., 2007).

### Tight Junction Molecules in the NFL

The NFL was clearly positive for claudin-1, which is considered a ubiquitous TJ protein occurring in most body tissues (Furuse et al., 1998). Claudin-11 is a specific component of TJ strands between myelin lamellae formed by oligodendrocytes in the (mammalian) CNS (Morita et al., 1999). Recent studies showed that claudin-11 functions as a diffusion barrier rather than providing mechanical stability between adjacent layers of myelin (Denninger et al., 2015). Consequently, claudin-11 null mice showed increased current flow across myelin sheaths resulting in mild tremors and hind limb weakness, although the myelin ultrastructure was unaltered. Claudin-19 is the main claudin expressed in peripheral nerve myelin formed by Schwann cells (Miyamoto et al., 2005). Besides being detected in the fish retina by PCR, the immunostains showed strong reactivity for claudin-19 in the retinal NFL. Thus, although claudin-19 in mammals is restricted to peripheral myelin, it is present in CNS of fish.

Claudin-3 and -5 are known for their presence in brain endothelial cells contributing to the blood-brain barrier (Wolburg et al., 2009). Claudin-3 has been suggested to be involved in the formation of new TJs, e.g., in endometrial cells during blastocyst attachment (Schumann et al., 2015), and at new cell contacts between Sertoli cells (Smith and Braun, 2012). The detection of claudin-3 in the retinal NFL may therefore reflect the ongoing restructuring of TJs due to continued growth of the retina. For claudin-5, two different orthologs of the human gene have been reported to be expressed in the zebrafish embryo. Claudin-5a was detected in the developing central nervous system of zebrafish, with a particularly strong expression in the neuroepithelial cells lining the brain ventricles whereas claudin-5b is considered endothelial-specific (Zhang et al., 2010). PCR results showed that both claudin genes are expressed in the cichlid retina, as well as in brain tissue. If claudin-5a plays a role in brain morphogenesis as suggested by Zhang et al. (2010), it might also be important for the facilitation of continued growth in the fish retina. The ortholog gene of the zebrafish claudin-k, expressed by oligodendrocytes, has been reported as claudin-31 in other fish species (Münzel et al., 2012; Baltzegar et al., 2013). We found that the predicted sequence designated claudin-9-like for *A. burtoni* was closely related to claudin-k, and demonstrated the expression of this tight junction protein in the fish retina by PCR analysis. However, gene expression comparisons are problematic because of the high number of claudins in fish (> 50), and the confusion in nomenclature and homology of claudins between different species (Baltzegar et al., 2013), e.g., zebrafish claudins are designated both alphabetically and numerically.

### Glial Cells in the NFL

The NFL contains the axonal nerve fibers of retinal GCs and the radial processes of Müller glial cells forming endfeet at the inner limiting membrane. In addition, in mammalian retinas with blood vessels, astrocytes are present in the NFL (Schnitzer, 1988a,b). Thus it is conceivable that Müller cell processes or astrocytes form the TJs in the NFL. The immunostains for axons together with claudin-1 and nuclear labeling suggest that the nuclei in the NFL most likely belong to the cells expressing claudin-1. This is supported by previous observations in zebrafish that cells positive for claudin-k expressed o*lig2*, a marker for oligodendrocytes (Münzel et al., 2012) suggesting the presence of oligodendrocytes inside the zebrafish retina. Furthermore, cell bodies in the NFL were negative for GFAP in the cichlid (see **Figure 1**). This suggests the cells identified in this layer are not astrocytes but a type of oligodendrocyte, and the probable source of loose-myelin wraps that had been clearly identified by electron microscopy. Since the loose wrap myelin differs structurally and molecularly (no MBP) from brain myelin, we call these cells oligodendroglia-like cells. Recently, these cells were shown to express Sox10 in the NFL of the goldfish retina (Parrilla et al., 2016).

The evolution of myelin has been linked to the presence of MBP (Werner, 2016). This raises the question whether the lack of MBP in loose wraps represents an evolutionary old form of myelin or oligodendroglial cells. Marques et al. (2016) revealed a diversity of oligodendrocytes in the juvenile and adult mouse brain although most of them belong to different stages of oligodendrocyte maturation. Besides the claudins mentioned above, molecular differences between peripheral and central myelin have been known for some time in mammals, for example P0 glycoprotein is present in the PNS and proteolipid protein (PLP) in the CNS. However, the distribution is different in aquatic species where P0-like proteins occur in PNS and CNS (Inouye and Kirschner, 2016; Zalc, 2016). In fact, the occurrence of PLP in myelin is considered to have emerged at the root of tetrapods (Möbius et al., 2009) although the gene appears to be present in fish (Brösamle, 2010). This is consistent with our finding that the oligodendroglial-like cells were negative for PLP-1 although immonoreactivity was present in the retina. It is noteworthy that not all Schwann cells and oligodendrocytes from myelin sheaths around associated axons. For example, so-called satellite oligodendrocytes in mammalian cortical gray matter were thought to be non-myelin forming oligodendrocytes but might be recruited during re-myelination (Ludwin, 1979). A more recent study suggests that these cells are part of a glial syncytium interacting with neuronal activity (Battefeld et al., 2016). Thus, the role of some oligodendrocytes is not merely restricted to form myelin wrappings but can be involved in other functions.

### Functional Role of TJs in the NFL

The presence and unique structure of TJs in the NFL raises the question of their significant function. In mammals, myelination in the visual pathway starts at the optic nerve head. The reason not to have myelin in the retina is not well understood, yet one explanation for this might be the strong light diffraction caused by dense myelin lamellae impairing vision. The loose wrapping of myelin in the fish retina might therefore be a less compromising way to ensheath axons without interfering with the optics. However, how much the sparse wrappings observed in some electron microscopy images serve a faster signal conductance is unknown.

We hypothesized that the loose myelin wraps and membrane faces with TJs in the NFL may function as a barrier that separates the vitreal milieu from the retinal compartment in the retina. Experiments with Evans Blue showed retention of the dye in the NFL before reaching the inner and outer layers. This suggests a barrier function by the existing TJs. Since the endfeet of Müller cell at the basal lamina of the inner limiting membrane do not form barrier junctions, the observed NFL TJs might contribute to limiting diffusion in and out of the retina.

In this context, it is of interest that the retina of fish continues to grow throughout life (Müller, 1952; Lyall, 1957; Meyer, 1978). Thus, axons of newly formed GCs in the peripheral growth zone must navigate through the existing and functioning NFL to reach the brain. In addition, the capacity of the fish retina and the optic nerve to regenerate has been well documented (Attardi and Sperry, 1963; Hitchcock et al., 1992). We localized new axons by immunolabeling with doublecortin, a marker for maturing neurons (Brown et al., 2003) including fish retina (Fernández-López et al., 2016; Sánchez-Farías and Candal, 2016), and found that the new axons run at the vitreal side of the NFL surrounded only by Müller cell endfeet (**Figure 8**). The Müller cell endfeet contain high levels of the water channels AQP4, thought to be involved in homeostasis (Nagelhus et al., 1998; Bringmann et al., 2006), although the polarized expression is less pronounced or absent in fish (Grupp et al., 2010; Gleiser et al., 2016). Interestingly, AQP4 has been implicated previously to be involved in regulating ocular growth (Goodyear et al., 2009). Thus, the TJ in the NFL together with the Müller cell endfeet might contribute to a microenvironment facilitating axonal growth. This idea is consistent with the observations obtained in the Evans Blue experiments, but clearly more work is needed to provide evidence for this notion.

## Author Contributions

LG-P performed the PCR, most of the stainings, confocal microscopy, and wrote parts of the manuscript. CG and AW designed the PCR experiments, interpreted the PCR results, and edited the manuscript. HW was involved in the freeze-fracture preparation and interpretation. AM designed the experiments, performed electron and confocal microscopy, and wrote the manuscript.

## Conflict of Interest Statement

The authors declare that the research was conducted in the absence of any commercial or financial relationships that could be construed as a potential conflict of interest.
